# Associations between employee and manager gender: impacts on gender-specific risk of acute occupational injury in metal manufacturing

**DOI:** 10.1186/1471-2458-13-1053

**Published:** 2013-11-08

**Authors:** Jessica T Kubo, Mark R Cullen, Manisha Desai, Sepideh Modrek

**Affiliations:** 1 Quantitative Sciences Unit, Stanford University School of Medicine, Stanford, CA, USA; 2 Division of General Medical Disciplines, Department of Medicine, Stanford University School of Medicine, Stanford, CA, USA

**Keywords:** Injury, Management, Sex, Gender concordance, Occupational health

## Abstract

**Background:**

Prior research has shown increased risk of injury for female employees compared to male employees after controlling for job and tasks, but have not explored whether this increased risk might be moderated by manager gender. The gender of one’s manager could in theory affect injury rates among male and female employees through their managers’ response to an employee’s psychosocial stress or through how employees differentially report injuries. Other explanations for the gender disparity in injury experience, such as ergonomic factors or differential training, are unlikely to be impacted by supervisor gender. This study seeks to explore whether an employee’s manager’s gender modifies the effect of employee gender with regards to risk of acute injury.

**Methods:**

A cohort of employees and managers were identified using human resources and injury management data between January 1, 2002 and December 31, 2007 for six facilities of a large US aluminum manufacturing company. Cox proportional hazards models were employed to examine the interaction between employee gender and whether the employee had female only manager(s), male only manager(s), or both male and female managers on injury risk. Manager gender category was included as a time varying covariate and reassessed for each employee at the midpoint of each year.

**Results:**

The percentage of departments with both female and male managers increased dramatically during the study period due to corporate efforts to increase female representation in management. After adjustment for fixed effects at the facility level and shared frailty by department, manager gender category does not appear to moderate the effect of employee gender (p = 0.717). Manager category was not a significant predictor (p = 0.093) of time to first acute injury. Similarly, having at least one female manager did not modify the hazard of injury for female employees compared to males (p = 0.899) and was not a significant predictor of time to first acute injury (p = 0.601).

**Conclusions:**

Prior findings suggest that female manufacturing employees are at higher risk for acute injury compared to males; this analysis suggests that this relationship is not affected by the gender of the employee’s manager(s).

## Background

The demographics of the US workforce continue to change with respect to women [1]. Women’s labor force participation is projected to increase 7.4% between 2010–2020 compared to 6.3% for men in the same period [2]. The changing composition of women in the workforce is reflected in management where there has also been a marked increase. The percent of women in management in the private sector increased from 32.7% in 1998 to 36.9% in 2011 [3]. Our own research setting, the fabricated metal product manufacturing industry, has one of the lowest percentages of female managers; in 2012 16.5% of industrial production managers were female [4].

As the number of women in managerial roles increases, particularly in the manufacturing sector, understanding the consequences on worker outcomes becomes increasingly necessary. Previous studies suggest that managers and management are a major determinant of workplace safety culture [5]; however, prior research focuses largely on men and does not consider the impact of manager gender. Of particular interest is worker injury because women employees experience a higher rate of injury for comparable job titles in many industries [6-10]. In a cohort of heavy manufacturing employees, women were found to have a 37% increase in risk compared to men [6]; female postal workers in their first year of employment had nearly double the risk of male workers (RR 1.93) [7]. In semiconductor manufacturing, women had a higher incidence rate for injury and illness than men [8]. Among utility workers, the rate ratio for injury for females compared to males was 1.5 [9] with the effect persisting in analyses of more severe injuries.

There have been several hypothesized reasons for the higher rate of injury amongst women including 1) a lack of tailoring of workplaces for female ergonomics and ill-fitting safety equipment [11] 2) a lack of formal job training and/or informal training through childhood activities and past jobs compared to men [9], 3) differential injury reporting, where men and women report injuries differently or experience injuries differently, particularly mild injuries [7,12], 4) social isolation (whereby women feel that they cannot ask for help when necessary), and 5) work-family conflict [13].

While the first two pathways, lack of tailoring of equipment or childhood experience, are unlikely to be related to the gender of an employee’s manager, there could be pathways whereby manager gender mediates women’s injuries through each of the last three mechanisms or through some combination of these. First, women may appear to have more injuries because of differences in reporting; reporting may potentially differ by manager gender or by whether employee and manager gender are concordant or discordant. Second, if a female employee is discordant with her colleagues and manager, she may be isolated and more at risk of injury. Third, it is possible that female employees have different psychosocial strains such as family responsibilities and responsibility for child care than male employees. Consequently the differences in management style, particularly styles related to gender norms, might help buffer against these psychosocial strains. Overall, it is *ex ante* ambiguous what effects an employee’s manager’s gender might have on the increased risk of injury experienced by female workers.

This study is an exploratory analysis of the effect of manager gender. In particular, we seek to explore whether an employee’s manager’s gender moderates the effect of employee gender with regards to risk of acute injury.

## Methods

### Study population

The study population includes 5,239 light manufacturing workers of a global aluminum company at six U.S. facilities employed during the period from January 1, 2002 through December 31, 2007. We select workers in the light manufacturing segment of the company because this sector employs a large proportion of female employees; other sectors did not have sufficient numbers of female managers to include in this analysis. The data were obtained from sources described in previous publications [6,10,14-16]. Briefly, a comprehensive real-time incident management system requires recording of all first-aid and reportable injury events. This database is linked to administrative human resource, health, work-environment and socio-demographic databases for research purposes. Employees from departments in which the majority of workers were salaried or from departments with less than ten employees were not included in the analytic cohort. Further, we did not include departments for which there was no clearly defined manager. The analytic cohort consists of 4,967 hourly employees and 272 managers across 99 departments.

### Identification of managers and manager gender category

An employee with a recorded job title suggestive of a leadership role (examples include “leader”, “manager”, “foreman” and “supervisor”) was considered to be a manager. Managers were defined at the midpoint of each year (July 1) and manager type was defined for all departments at this point. Departments were categorized based on whether they had female managers only, male managers only, or both female and male managers.

### Outcomes

The outcome of interest is the time from the start date of an employee in a new department to his or her first acute injury. Employees who worked in more than one department during the study period may be included in the risk set more than once; however, repeated injuries within the same department are not considered as the majority of injuries in this cohort were first injuries (>70%). Injury outcomes were obtained from the incident management system. Only acute injuries, such as burns, lacerations, contusions and fractures, were considered.

### Statistical methods

Cox proportional hazards models were used to model time to first acute injury. Employees were censored administratively on December 31, 2007, when they quit or were terminated, or when they changed departments, whichever occurred first. As we expect correlation among employees within the same department, we employed shared frailty models to adjust for correlation of injury risk. The shared frailty model incorporates a random effect for each department and assumes that these terms follow a gamma distribution [17-19].

Manager type (female manager only, male manager only, or both male and female managers) was defined each year for all employees and was included as a time-varying covariate. Manager type was defined for each department on July 1 of each year; employees were assigned a manager type in each year based on the department they were in on July 1. The predictor of interest was the interaction between employee gender and manager type category.

At the department level, we defined departments where the physical demands of the work was likely to be particularly high using the most common job titles within the department. If one or more of the three most common job titles within a department contained the words “metal,” “heat,” “foundry,” “welder,” “furnace,” or “kiln,” the department was defined as having jobs that required particularly high physical demand. This variable is important to include in the analyses because physical demand is related to injury risk and there are more male employees and male managers in high demand departments.

In the primary analyses, we fit three models: an unadjusted model (Model 1) adjusted for time-varying manager gender type, employee gender, and the interaction term, an adjusted model that additionally adjusted for employee race/ethnicity, employee age, employee tenure at the company, and whether the department included jobs that required high physical demand or not (Model 2), and an adjusted model that additionally included a frailty term at the department level (Model 3). All models were adjusted for fixed effects at the facility level. To address concerns about the presence of a secular trend in injuries during the study period, we further adjusted for calendar year as a time-varying covariate in all models. Employees who were ever managers during the study period were excluded from modeling.

In secondary analyses, we categorized departments as having at least one female manager or having no female managers and explored the interaction between having a female manager and the employee’s gender. We also explored the issue of discordance, i.e. male employees with female managers only and female employees with male managers only compared to all other employee-manager pairings. While only medical treatment, restricted work, and lost work time injuries are reportable to OSHA, the majority of injuries in this cohort were first aid only. Most studies of injury only include OSHA-reportable injuries (as they are likely the only ones recorded in employee databases) and do not track first-aid injuries though these types of injuries are likely very different. In secondary analyses we examined these categories of injury as outcomes separately.

**Figure 1:**
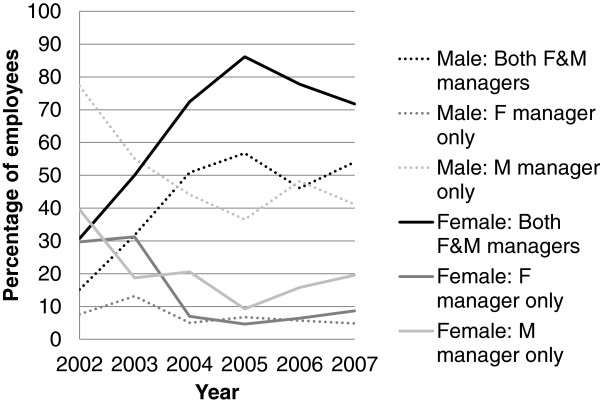
Manager gender category of the cohort for male and female employees by calendar year.

To ensure the robustness of our results we conducted several sensitivity analyses. First, results from Cox models were compared to parametric survival models utilizing Gompertz and Weibull distributions to assess whether the choice of a proportional hazards model was reasonable. Second, to further examine the potential pathway of work-family conflict, we explored including having a dependent child under the age of 6 as a possible mediating variable. Third, we explored the previous year’s manager type instead of the current year’s manager gender category to address the issue of employees changing managers and/or departments in response to an injury. Finally, since the percentage of employees with each manager gender category changed dramatically during the early study period (see below), we performed a sensitivity analysis using only employee-departments starting after January 1, 2004 when manager gender categories had stabilized (Figure 1).

Analyses were performed with SAS software, Version 9.3 (SAS Institute Inc., Cary, NC) of the SAS System for Windows and Stata 12 (StataCorp, College Station, TX).

The protocol was approved, invoking the epidemiologic exemption waiving the requirement for individual consent, by the Stanford University IRB.

## Results

Table 1 displays characteristics and demographics of all employees by gender. Fewer female employees were in departments classified as high demand compared to males (19.8% vs. 36.16%). A higher percentage of female employees were white. Females were older when entering new departments, with longer tenures than males (mean age 43.5 vs. 39.8; mean tenure 8.6 years vs. 7.3 years). A higher percentage of females started in departments led by both female and male managers compared to males (54.6%), more male employees were in departments led by male managers only (53.6%).

**Table 1:** Baseline demographics of analysis cohort by employee gender

**Characteristic**	**Female employees**	**Male employees**	**Total**	**P-value**
*Total (N, percent)*	2,322	2,645	4,967	
	46.75	53.25		
*Employee race/ethnicity (N, percent)*				<.001
American Indian	10	16	26	
	0.43	0.60		
Asian	43	54	97	
	1.85	2.04		
Black	394	585	979	
	16.97	22.12		
Hispanic/Latino	304	372	676	
	13.09	14.06		
White	1,571	1,618	3,189	
	67.66	61.17		
*Age when started in department by employee-department (mean, SD)*	43.53	39.81	41.68	<.001
	11.08	11.39	11.38	
*Tenure at company when started in department by employee-department (mean, SD)*	8.63	7.27	7.96	<.001
	8.98	9.15	9.09	
*High demand department by employee department (N, percent)*				<.001
Not high demand	3,495	2,740	6,235	
	80.25	63.84		
High demand	860	1,552	2,412	
	19.75	36.16		
*Manager gender when started in department by employee department (N, percent)*				<.001
Both female and male	2,379	1,561	3,940	
	54.63	36.37		
Female only	1,045	431	1,476	
	24.00	10.04		
Male only	931	2,300	3,231	
	21.38	53.59		

Note: P-values are from a Chi-square test of association for categorical variables and from a t-test for continuous variables.

**Figure 2:**
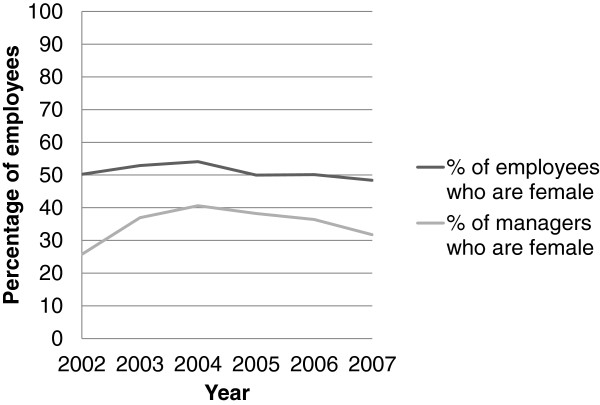
Gender of employees in the cohort by calendar year.

Figures 1 and 2 present percentages of male and female employees in each category of manager type by year over the study period and percentages of female employees by year over the study period respectively. During this period, the percentage of employees who had both female and male managers doubled, with corresponding drops in employees with female managers only and male managers only. Very few male employees were in departments for which all managers were female; this percentage stayed fairly constant at about 6% for most of the study period.

The 99 departments included had an average count of 51.7 employees (SD 61.2, median 29). Of these, 30 (30.3%) were considered high physical demand based on job titles within the department.

Over a mean follow up of 4.86 years (SD 3.64), 683 employees in departments with male and female managers were injured (17.34%), 180 employees in departments with female managers only were injured (12.20%) and 453 employees in departments with male managers only were injured (14.02%). Characteristics of first acute injuries during the study period by employee gender are shown in Table 2. The vast majority of injuries were first aid injuries, which are not reportable to OSHA (83.5%); a higher percentage of injuries among female employees were first aid (87.9%) compared to male employees (78.7%). Injury severity and type were significantly associated with employee gender (p < 0.001 and p < 0.001 respectively). Contusion/bruise was the most common injury type among female employees and laceration/cut was the most common injury type among male employees.

**Table 2:** Description of first acute injuries in the cohort

	**Female employees**		**Male employees**	
**Injury characteristic**	**Count**	**Percent**	**Count**	**Percent**
*Total*	687		629	
*Severity (Chi-square p < .001)*				
First aid (non-OSHA-reportable)	604	87.92	495	78.70
Medical treatment	37	5.39	62	9.86
Restricted work	44	6.40	67	10.65
Lost work time	2	0.29	5	0.79
*Type of injury (Chi-square p < .001)*				
Laceration/Cut	133	19.36	183	29.09
Contusion/Bruise	169	24.60	100	15.90
Instantaneous sprain/Strain	73	10.63	92	14.63
Burn (chemical or thermal)	97	14.12	48	7.63
Abrasion/Scratch	63	9.17	74	11.76
Foreign body	57	8.30	71	11.29
Other	95	13.83	61	9.70

**Figure 3:**
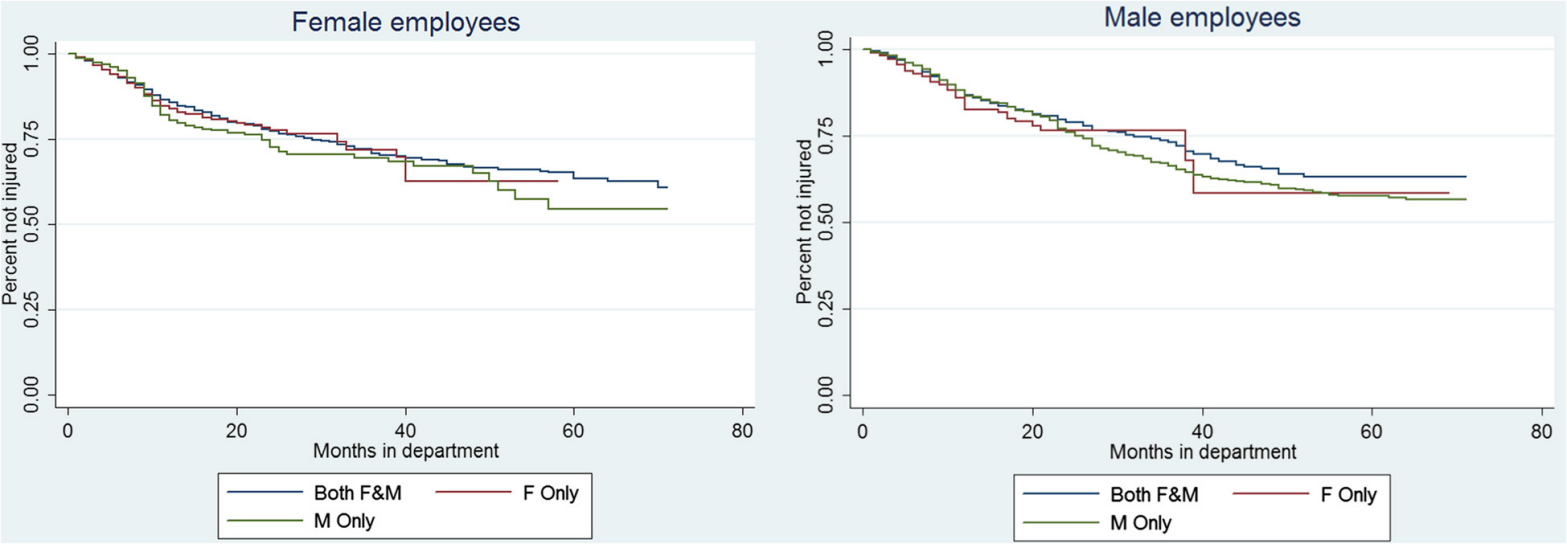
Kaplan-Meier estimates by manager gender category for female (left) and male (right) employees.

Figure 3 presents Kaplan-Meier survival estimates by manager gender category for female and male employees separately. The descriptive plots show that by 3 years (36 months) over 25% of both male and female employees experience an injury, but there does not seem to be a marked difference by manager gender. In Table 3 these relationships are examined in a multivariate manner using Cox proportional hazards models. In all three models, the Wald p-value for the interaction between employee gender and manager gender category was not significant and the interaction was excluded from models in Table 3. Results from the interaction models are included in the Additional file 1: Table S1. In the adjusted model including a frailty term at the department level (Model 3), female gender was associated with a HR of 1.21 (CI 1.06 – 1.39) compared to male gender for injury risk. Manager gender category was not significantly associated with injury risk (p = 0.093) when comparing both female and male managers and only female managers to only male managers.

**Table 3:** Results from models of employee gender and manager type on time to first acute injury

**Covariate**	**Unadjusted model**		**Adjusted model**		**Model including shared frailty at department level**	
	**HR (95% CI)**	**p-value**	**HR (95% CI)**	**p-value**	**HR (95% CI)**	**p-value**
*Gender*		0.042		0.054		0.006
F vs. M	1.13 (1.00, 1.28)		1.13 (1.00, 1.28)		1.21 (1.06, 1.39)	
*Manager type (time-varying)*		0.324		0.274		0.093
Both vs. M only	0.93 (0.80, 1.07)		0.96 (0.83, 1.11)		0.97 (0.79, 1.19)	
F only vs. M only	1.04 (0.86, 1.26)		1.10 (0.91, 1.34)		1.25 (0.97, 1.60)	
*Interaction*	Not included in final model	0.225	Not included in final model	0.519	Not included in final model	0.717
*Location*	Not shown	<.001	Not shown	<.001	Not shown	<.001
*Year (time-varying)*	Not shown	<.001	Not shown	<.001	Not shown	<.001
*Race/Ethnicity*				0.014		0.016
Am. Indian vs. White			0.98 (0.51, 1.89)		0.97 (0.50, 1.88)	
Asian vs. White			0.46 (0.28, 0.75)		0.46 (0.28, 0.75)	
Black vs. White			0.97 (0.81, 1.17)		0.97 (0.81, 1.17)	
Hispanic vs. White			1.11 (0.93, 1.32)		1.11 (0.93, 1.32)	
*Age when started in dept.*			0.99 (0.99, 1.00)	0.020	0.99 (0.99, 1.00)	0.013
*Tenure when started in dept.*			1.00 (0.99, 1.00)	0.359	1.00 (0.99, 1.00)	0.393
*Department is high demand*			0.80 (0.69, 0.92)	0.001	0.77 (0.61, 0.97)	0.025

### Secondary analyses

The results from models exploring departments with any female manager and those with no female manager were similar to the models in which manager type was separated into three categories. Again, the interaction between manager type and employee gender was not significant (p = 0.899) and females had an elevated HR of 1.33 (CI 1.16, 1.54) compared to males for time to first injury. Hazard of injury was not significantly different for employees having at least one female manager and those who did not have a female manager (p = 0.601). Discordant pairs (male employees with only female managers and female employees with only male managers) did not significantly differ in hazard of injury compared to concordant pairs (HR 1.02 (CI 0.85, 1.24), p-value = 0.806) (Additional file 1: Table S2).

The majority of injuries in this cohort are first-aid only (83.6%) but as OSHA and company policy focus on medical treatment, restricted work, and lost work time injuries, we examined first-aid and reportable injuries separately. For time to first aid injury, manager gender category did not significantly modify the hazard for females compared to males (p = 0.952). In models without the interaction term, female employees had an increased hazard of injury compared to males (HR 1.31 (CI 1.14, 15.2), p-value < .001). Further, employees who had only female manager(s) had an increased hazard of injury (HR 1.37 (CI 1.06, 1.79), p-value 0.030) compared to employees who had only male manager(s).

A higher proportion of men experienced a reportable injury (6.05% compared to 4.61% of females). For time to reportable injury, the HR for female employees compared to male employees was not significantly different by manager gender category (p = 0.064) in the model including a shared frailty term at the department level (Table 4). Manager gender category was not significant (p = 0.496) in the model without the interaction term. In the same model, male employees had a borderline significant decrease in risk of reportable injury compared to females (HR 0.74 (CI 0.55, 1.00, p-value 0.05).

**Table 4:** Results from models of employee gender and manager type on time to first reportable acute injury and first first-aid acute injury

**Covariate**	**Model including shared frailty at department level**			
	**First-aid injury (83.6%)**		**Reportable injury (16.4%)**	
	**HR (95% CI)**	**p-value**	**HR (95% CI)**	**p-value**
*Gender*				
F vs. M	1.31 (1.14, 1.52)	<.001	0.74 (0.55, 1.00)	0.049
*Manager type (time-varying)*		0.030		0.496
Both vs. M only	1.03 (0.83, 1.27)		0.83 (0.58, 1.19)	
F only vs. M only	1.37 (1.06, 1.79)		0.77 (0.46, 1.30)	
*Interaction*	Not included in final model	0.952	Not included in final model	0.064
*Location*	Not shown	<.001	Not shown	0.295
*Year (time-varying)*	Not shown	<.001	Not shown	<.001
*Race/ethnicity*		0.058		0.442
Am. Indian vs. White	0.93 (0.46, 1.88)		0.66 (0.09, 4.73)	
Asian vs. White	0.49 (0.30, 0.81)		0.34 (0.08, 1.38)	
Black vs. White	0.99 (0.82, 1.21)		0.94 (0.64, 1.39)	
Hispanic vs. White	1.08 (0.89, 1.29)		1.21 (0.80, 1.82)	
*Age when started in dept.*	0.99 (0.99, 1.00)	0.013	0.99 (0.87, 1.01)	0.412
*Tenure when started in dept.*	1.00 (0.99, 1.01)	0.493	1.00 (0.98, 1.01)	0.546
*Department is high demand*	0.75 (0.59, 0.96)	0.024	0.71 (0.49, 1.02)	0.063

### Sensitivity analyses

We conducted several sensitivity analyses to ensure the robustness of our results. To ensure that our results were not dependent on the distributional properties of the model, we compared parameter estimates from Cox models to those using Gompertz or Weibull models. Results and interpretation from the Cox proportional hazards models did not differ from those obtained using Gompertz and Weibull parametric survival models. Second, we examined the role of children in injury risk. Having a dependent child under the age of 6 was not significant in our final specification, Model 3, and excluding it from the model did not change point estimates for the variables of interest. To address the issue of employees changing managers and/or departments in response to an injury, we examined the timing of supervisor gender with regard to injury. Results and interpretation did not change in the analysis using the previous year’s manager type in place of current manager gender category. Second, to ensure that we do not over weight the transition period when the gender mix of managers was changing drastically, we restricted the analyses to employee-departments starting after January 1, 2004. This too did not change results and interpretation.

## Discussion

Our analysis of light manufacturing employees at six facilities of a major US aluminum company does not suggest that the increased hazard of acute injury associated with female gender is modified by the employee’s manager’s gender.

The inability to demonstrate any benefit in terms of the gender disparity in injury risk consequent to a major ramp-up in the gender mix of supervisors sheds some light on the cause or causes of the disparity: Either gender-match is insufficient to buffer the social causes such as isolation, management of personal family conflict or differential reporting, or those causes are less salient than ergonomic differences between men and women, or differences in their overall training. While there are undoubtedly numerous reasons to expand the numbers of women in these traditional male supervisory roles, reduction of female injury risk does not appear to be a strong one.

Our analysis found that female employees had a 20% increased risk of injury compared to male employees. This is similar to the finding by Taiwo et al. in heavy manufacturing workers at aluminum smelters where female employees had an increased risk of 37% compared to male employees [6]. Similar results have been found in other settings. Kelsh et al. found that after adjusting for occupation, job experience and age, female electric utility workers had a higher rate of injury compared to males [9]. Similarly, higher incidence of injury among females was observed in a cohort of semiconductor manufacturing workers by McCurdy et al. [8]. Buchanan et al. found that female hotel employees were injured more frequently than male employees; the majority of housekeepers, who experience high physical demand, are women [20].

Surprisingly, being in a high demand department was consistently associated with a reduction in hazard of acute injury in this subset of plants. A possible explanation for this is the healthy worker effect, i.e. those who are injury-prone are selected out of high demand jobs. Further, departments designated as high demand contained a much higher percentage of male workers than non-high demand departments and employees in these departments had significantly longer tenure in their department. “High demand department” may be reflective of factors we were unable to capture, such as tenure at the company, skill level, experience with a particular task, and dynamic selection into and attrition out of high departments based on previous injury experience and/or safety culture [21,22].

Secondary analyses using presence or absence of a female manager in the department support our primary result and suggest that the increased risk for female employees compared to male employees does not differ for departments with and without any female managers.

Results from outcomes restricted to first aid-only injuries and reportable injuries only did not differ from the main analysis for the research question exploring employee and manager gender concordance; however, reportable injuries make up a small fraction of injuries in the light manufacturing facilities we studied therefore we cannot be sure whether our results reflect a lack of power or a true null relationship between manager and employee gender. Of note, while a higher proportion of females experienced a first aid injury compared to males, the relationship is opposite for reportable injuries with a higher proportion of males experiencing a reportable injury compared to females. This is likely to do with differing potential for serious injury based on higher demand jobs, which are mostly done by men.

Further, we find some evidence that for first-aid injuries, both men and women employees have a higher risk of injuries when in a department led by female managers only compared to those in departments led by male managers only. This may be due to reporting of injuries by employees or by managers, or be due to the increase in female managers occurring during the study period – freshman managers have less experience in their department and may be more likely to report first-aid injuries than experienced managers.

A sensitivity analysis that used the previous year’s manager gender category was not different from the main analysis, suggesting that the effect of employees changing managers or departments in response to an injury is minimal. Sensitivity analyses that restricted analysis to employees starting in departments after Jan. 1, 2004, after the percentages of employees in each category of manager gender had stabilized, suggest that the effect of the change in manager demographics from 2002–2004 was minimal.

Strengths of this study include use of a cohort of manufacturing employees with a large number of female employees and female managers. Data, including injury and employment outcomes, were obtained from human resources records and an injury management database and not from self-report. Further, the real-time injury management database allowed us to examine first aid (non-reportable) injury as an outcome; this sector experiences few reportable injuries in proportion to total injury and this further allowed us to explore the issue of differential reporting.

This study does have important limitations. First, we were unable to distinguish floor managers from senior management when identifying managers. However, it has been suggested that both floor managers and senior management are very important to safety culture [5]. Another limitation is that men and women with the same job title may perform very different tasks [11], and that women are more likely than men to perform tasks involving repetitive motion and standing for extended periods [23]. Unmeasured confounders, such as work culture, that are associated with manager gender and injury as well as employment outcomes exist.

Given that only 17% of the employees were in departments with a discordant manager type (only female managers for a male employee and only male managers for a female employee), lack of power may have affected our ability to find a significant interaction between manager and employee gender. However, 13% of injuries were to discordant employees. We did not control for the number of hours worked, or overtime hours, and do not incorporate data on injuries after the first. Further, individuals may self-select into departments based on the gender of their manager, a factor we are unable to capture; however, this is unlikely to be the case in a period of rapidly changing manager composition. Perhaps most important of all, our dataset derives from a single industry and single safety-conscious employer, and hence is not necessarily representative of injury risks or their causes more generally.

## Conclusion

As our analysis was exploratory in nature, these results can be used to inform future analyses of effect modification of risk of injury for men and women by their manager’s gender as well as future analyses exploring pathways for the increased risk of occupational injury for females compared to males. As the percentage of women in management increases, especially in previously male-dominated industries, it is important to consider the impact of manager gender on injury and employment outcomes. In this cohort of light manufacturing workers, manager gender does not appear to modify the increased risk of acute injury experienced by female employees overall.

## Competing interests

Dr. Cullen serves as a senior medical advisor to Alcoa under the terms of a research contract between Stanford University and Alcoa, Inc.

The other authors have no competing interests to report.

## Authors’ contributions

JK participated in designing the research plan, performed all statistical analyses and co-wrote the manuscript. MRC and MD participated in designing the research plan, provided feedback on the analyses, and edited the manuscript. SM participated in designing the research plan, provided feedback on the analyses, and co-wrote the manuscript. All authors read and approved the final manuscript.

## Pre-publication history

The pre-publication history for this paper can be accessed here:

http://www.biomedcentral.com/1471-2458/13/1053/prepub

## Supplementary Material

Additional file 1: Table S1 Results from the model including an interaction between employee gender and manager gender. **Table S2.** Results from model comparing discordant and concordant employee-manager pairings. Click here for file
