# Supplementary material for: Associations between employee and manager gender: impacts on gender-specific risk of acute occupational injury in metal manufacturing
Source: BMC Public Health. 2013 Nov 8;13:1053. doi: 10.1186/1471-2458-13-1053 (PMC3924331; doi:10.1186/1471-2458-13-1053)
Supplement: Additional file 1: Table S1 — Results from the model including an interaction between employee gender and manager gender. Table S2. Results from model comparing discordant and concordant employee-manager pairings. [file 1471-2458-13-1053-S1.doc]

Table S1: Results from the model including an interaction between employee gender and manager gender

| **Covariable** | **Unadjusted model** | | **Adjusted model** | | **Model including shared frailty at department level** | |
| --- | --- | --- | --- | --- | --- | --- |
|  | HR (95% CI) | p-value | HR (95% CI) | p-value | HR (95% CI) | p-value |
| *Employee-Manager gender* |  | 0.225 |  | 0.519 |  | 0.717 |
| Female vs. male for Both managers | 1.20 (1.02, 1.41) |  | 1.16 (0.98, 1.37) |  | 1.26 (1.05, 1.50) |  |
| Female vs. male for Female only | 1.29 (0.93, 1.80) |  | 1.29 (0.93, 1.80) |  | 1.23 (0.88, 1.74) |  |
| Female vs. male for Male only | 0.97 (0.78, 1.21) |  | 1.03 (0.83, 1.29) |  | 1.11 (0.87, 1.42) |  |
| *Location* |  | <.001 |  | <.001 |  | <.001 |
| *Year (time-varying)* |  | <.001 |  | <.001 |  | <.001 |
| *Race/ethnicity* |  |  |  | 0.014 |  | 0.016 |
| Am. Indian  vs. White |  |  | 0.97 (0.50, 1.88) |  | 0.96 (0.50, 1.87) |  |
| Asian vs.  White |  |  | 0.46 (0.28, 0.75) |  | 0.46 (0.28, 0.75) |  |
| Black vs.  White |  |  | 0.97 (0.81, 1.17) |  | 0.97 (0.81, 1.17) |  |
| Hispanic vs.  White |  |  | 1.11 (0.93, 1.32) |  | 1.11 (0.93, 1.32) |  |
| *Age when started in dept.* |  |  | 0.99 (0.99, 1.00) | 0.019 | 0.99 (0.99, 1.00) | 0.012 |
| *Tenure when started in dept.* |  |  | 1.00 (0.99, 1.00) | 0.375 | 1.00 (0.99, 1.00) | 0.406 |
| *Department is high demand* |  |  | 0.81 (0.70, 0.93) | 0.003 | 0.77 (0.62, 0.98) | 0.031 |

Table S2: Results from model comparing discordant and concordant employee-manager pairings

| **Covariable** | **Unadjusted model** | | **Adjusted model** | | **Model including shared frailty at department level** | |
| --- | --- | --- | --- | --- | --- | --- |
|  | **HR (95% CI)** | **p-value** | **HR (95% CI)** | **p-value** | **HR (95% CI)** | **p-value** |
| *Employee-Manager gender* |  |  |  |  |  |  |
| Discordant (no manager of the same gender) vs. concordant | 0.95 (0.80, 1.13) | 0.545 | 0.98 (0.83, 1.16) | 0.831 | 1.02 (0.85, 1.24) | 0.806 |
| *Location* |  | <.001 |  | <.001 |  | <.001 |
| *Year (time-varying)* |  | <.001 |  | <.001 |  | <.001 |
| *Race/ethnicity* |  |  |  | 0.012 |  | 0.013 |
| Am. Indian  vs. White |  |  | 0.99 (0.51, 1.91) |  | 0.98 (0.51, 1.91) |  |
| Asian vs.  White |  |  | 0.46 (0.28, 0.74) |  | 0.46 (0.28, 0.75) |  |
| Black vs.  White |  |  | 0.97 (0.81, 1.16) |  | 0.97 (0.81, 1.16) |  |
| Hispanic vs.  White |  |  | 1.11 (0.94, 1.32) |  | 1.11 (0.94, 1.33) |  |
| *Age when started in dept.* |  |  | 0.99 (0.99, 1.00) | 0.044 | 0.99 (0.99, 1.00) | 0.042 |
| *Tenure when started in dept.* |  |  | 1.00 (0.99, 1.01) | 0.450 | 1.00 (0.99, 1.01) | 0.467 |
| *Department is high demand* |  |  | 0.78 (0.68, 0.90) | <.001 | 0.76 (0.61, 0.94) | 0.011 |
